# Supplementary material for: Structural and functional characterization of the Sin Nombre virus L protein
Source: PLoS Pathog. 2023 Aug 7;19(8):e1011533. doi: 10.1371/journal.ppat.1011533 (PMC10406178; doi:10.1371/journal.ppat.1011533)
Supplement: S1 Table — (DOCX) [file ppat.1011533.s001.docx]

| **DATA COLLECTION AND PROCESSING** |  |
| --- | --- |
| Magnification | 105,000 |
| Voltage (kV) | 300 |
| Electron exposure (e-/Å^2^) | 50 |
| Defocus range (µM) | -0.7 to -2.6 |
| Pixel size(Å) | 0.85 |
| Initial micrographs (no.) | 4283 |
| Selected micrographs (no.) | 2337 |
| Initial particles picked (no.) | 883,096 |
| Particles in the final reconstruction (no.) | 90,361 |
| Map resolution (Å) 0.143 FCS threshold | 3.2 |
| Map resolution range (Å) | 3 to 4.7 |
| **REFINEMENT** |  |
| Model resolution (Å) | 3.2 |
| FSC threshold | 0.5 |
| Map sharpening B factor (Å^2^) | -86 |
| Model composition: |  |
| Nonhydrogen atoms | 10366 |
| Protein residues | 1258 |
| Nucleotide residues | 11 |
| R.m.s deviations: |  |
| Bond lengths (Å) | 0.014 |
| Bond angles (°) | 1.186 |
| Validation: |  |
| MolProbity score | 1.68 |
| Clashscore | 3.74 |
| Poor rotamers (%) | 0.00 |
| Ramachandran plot: |  |
| Favored (%) | 91.11 |
| Allowed (%) | 8.32 |
| Disallowed (%) | 0.57 |
| PDB code | 8CI5 |
| EMDB code | EMD-16670 |
