# Supplementary figures and images for: Structural and functional characterization of the Sin Nombre virus L protein
Source: PLoS Pathog. 2023 Aug 7;19(8):e1011533. doi: 10.1371/journal.ppat.1011533 (PMC10406178; doi:10.1371/journal.ppat.1011533)

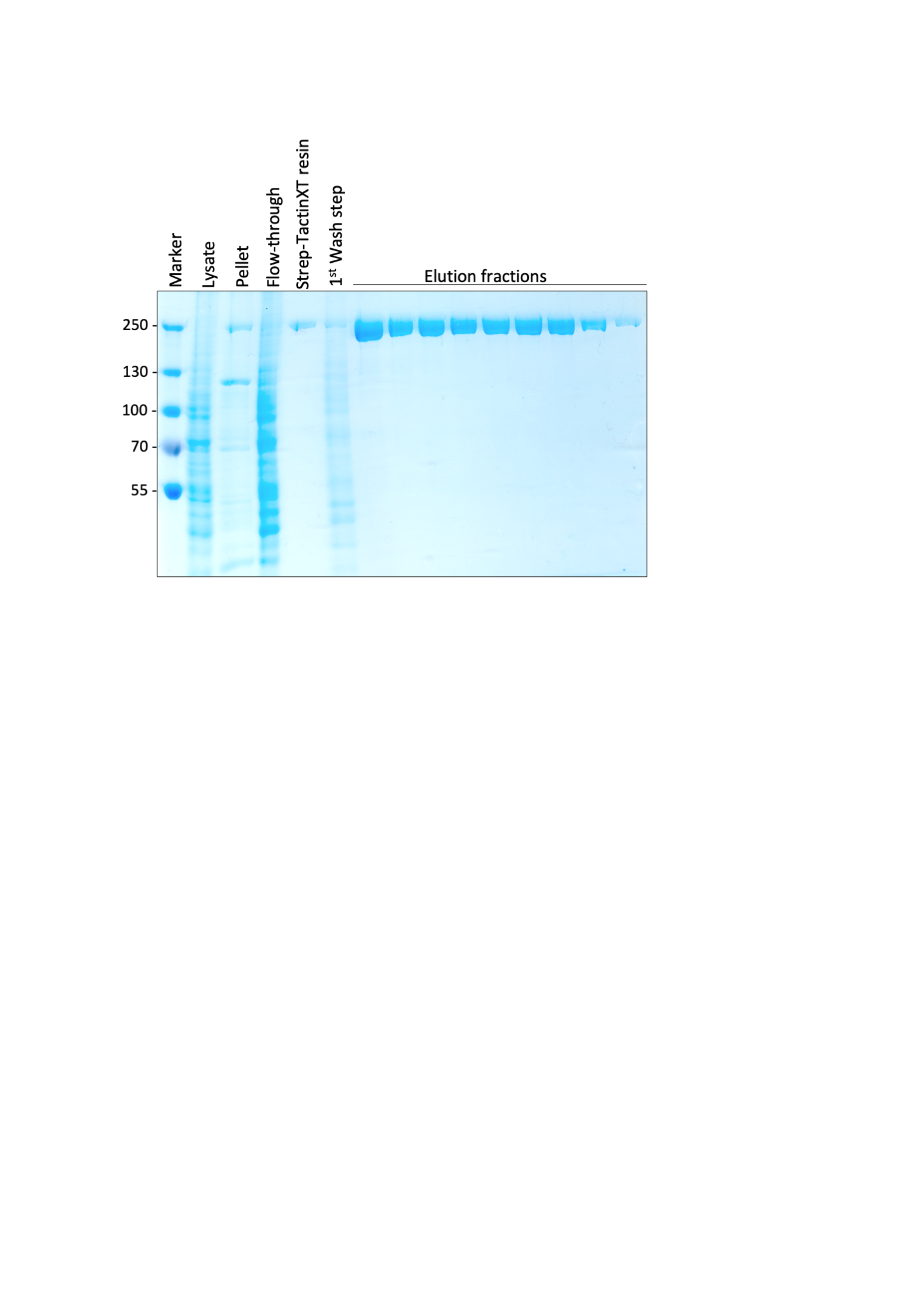

Supplement: S1 Fig — SNV L K124A with a C-terminal His6-StrepII-tag was purified using Strep-TactinXT resin (see Materials and Methods) and eluted in several fractions. Shown are samples of the Lysate, the pelleted unsoluble fraction, the flow-through, the resin with bound residual protein, the first wash step as well as the elution fractions with a molecular weight marker (PageRuler Plus Prestained, Thermo Scientific). The shown elution fractions were pooled and further purified via heparin chromatography (HiTrap Heparin HP, GE Healthcare) to remove any bound RNA. (TIFF) [file ppat.1011533.s002.tiff]

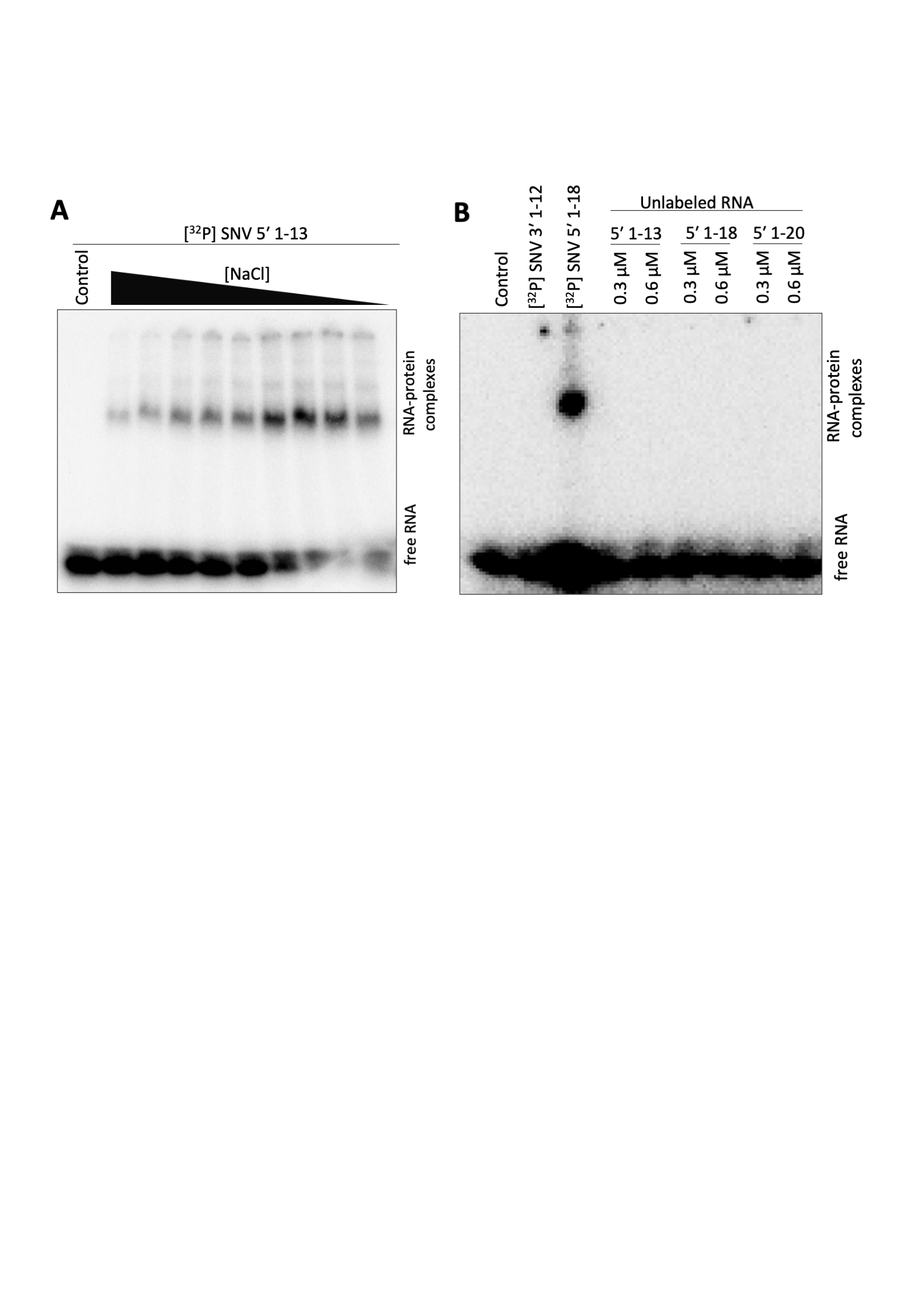

Supplement: S2 Fig — (A) Electromobility shift assay to determine the binding capacity of the SNV L protein to the 5′ RNA 1–13 in different salt concentrations. Radiolabeled 5′ RNA at a concentration of ~0.3 μM was incubated 0.2 μM L protein in EMSA buffer containing 500, 450, 400, 350, 300, 250, 200, 150, or 100 mM NaCl. The RNA-protein complexes were separated from free RNA by native PAGE and signals were visualized via phosphor screen autoradiography with a Typhon scanner (GE Healthcare). The control lane shows promoter RNA without L protein. (B) Electromobility shift assay to determine the recruitment of 3′ RNA 1–12 to the L protein in the presence of 5′ promoter RNA. Labeled 3′ RNA 1–12 at a concentration of ~0.3 μM was incubated with 0.2 μM L protein in the presence of unlabeled 5′ promoter RNA (nucleotides 1–13, 1–18, or 1–20) at an equimolar concentration or a two-fold excess of 5′ to 3′ RNA. Labeled 5′ RNA 1–18 was used as a positive control, a sample lacking L protein was used as a negative control. (TIFF) [file ppat.1011533.s003.tiff]

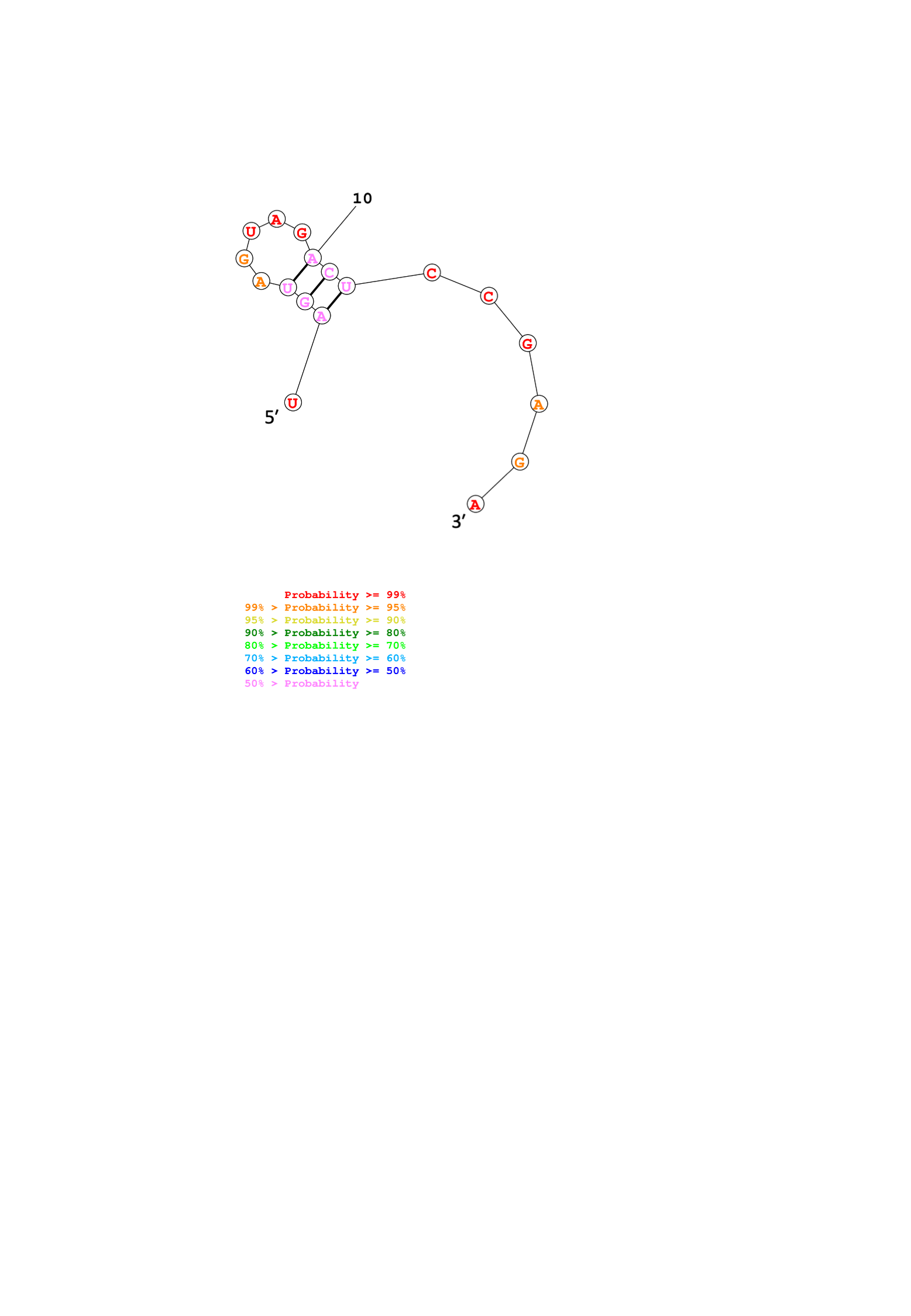

Supplement: S3 Fig — The secondary structure of SNV L 5 1–18 RNA (5‘-UAGUAGUAGACUCCGAGA-3‘) was carried out with RNAstructure [24]. (TIFF) [file ppat.1011533.s004.tiff]

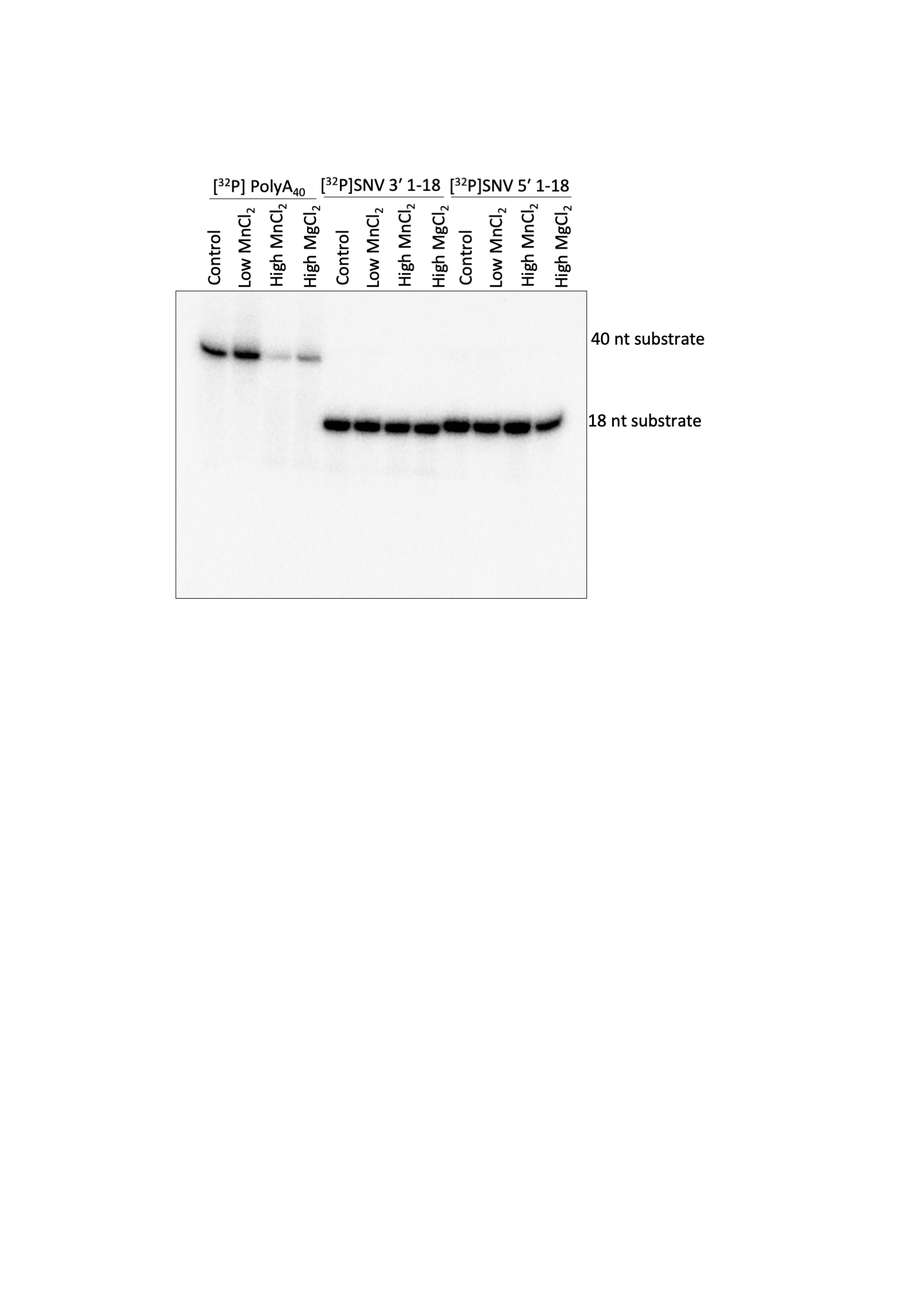

Supplement: S4 Fig — A 10 μL reaction containing 0.5 μM full-length SNV L K124A, radiolabeled RNA substrate, and 0.1 mM (low MnCl2) or 10 mM (high) MnCl2 or MgCl2 in reaction buffer was incubated at 37°C for one hour. The reactions were stopped by adding 10 μL of 2x RNA loading dye (98% formamide, 18 mM EDTA, 0.025 mM SDS, xylene cyanol, bromophenol blue) and heated to 95°C for five minutes. RNA was separated on denaturing PAGE (25% acrylamide, 7 M urea, 0.5 x TBE) and visualized by autoradiography. The control lane shows a reaction without the addition of SNV L K124A. (TIFF) [file ppat.1011533.s005.tiff]

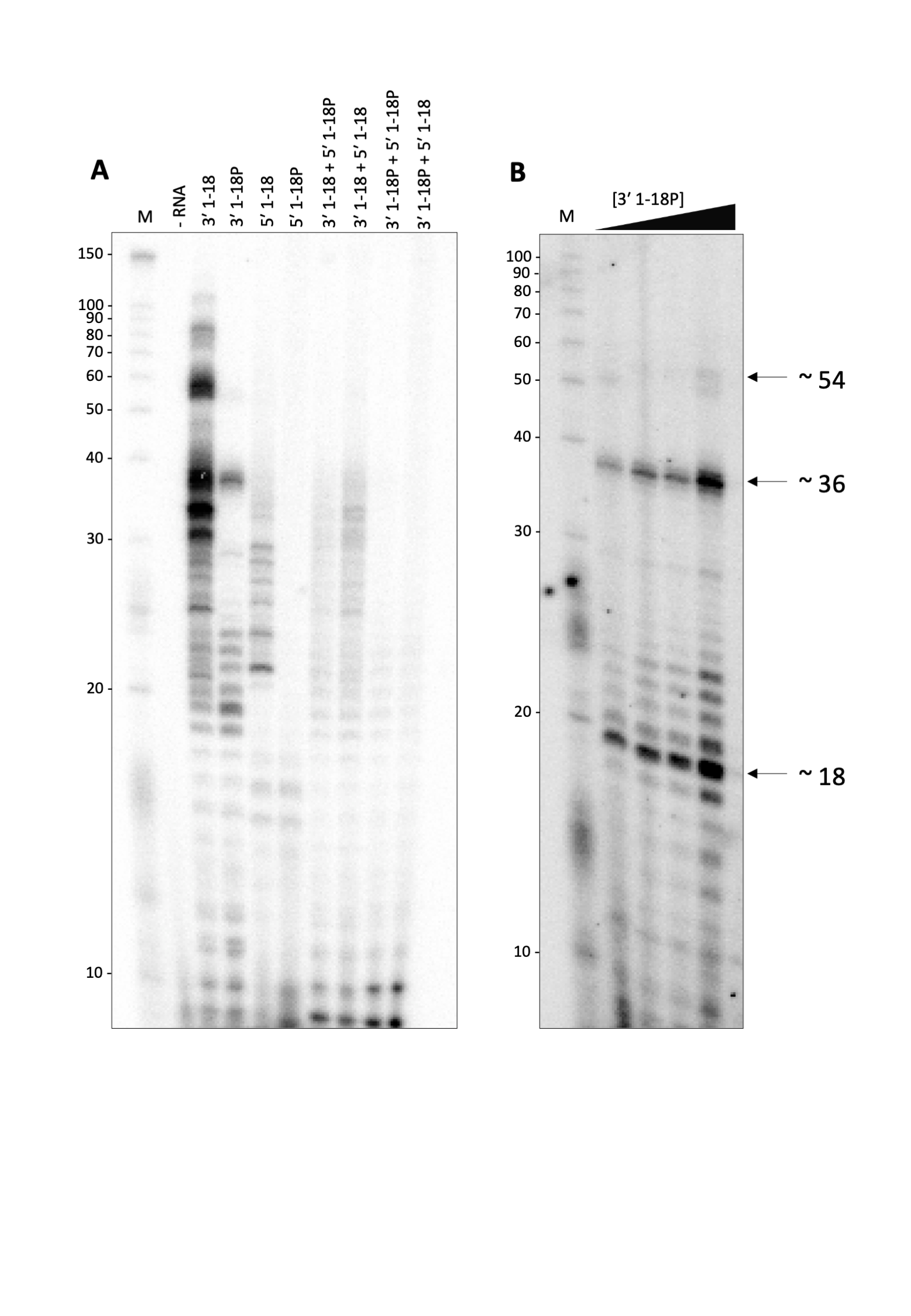

Supplement: S5 Fig — In a 10 μL reaction 4 pmol SNV L K124A was incubated with 6 pmol of the indicated RNA(s) (A) or increasing concentrations of SNV L 3′ 1-18P (6, 12, 20, or 40 pmol) (B). NTPs supplemented with [α]32P-GTP were added, and the reaction was incubated at 30°C for one hour. Reaction products were separated by denaturing PAGE and signals were visualized via phosphor screen autoradiography. (TIFF) [file ppat.1011533.s006.tiff]

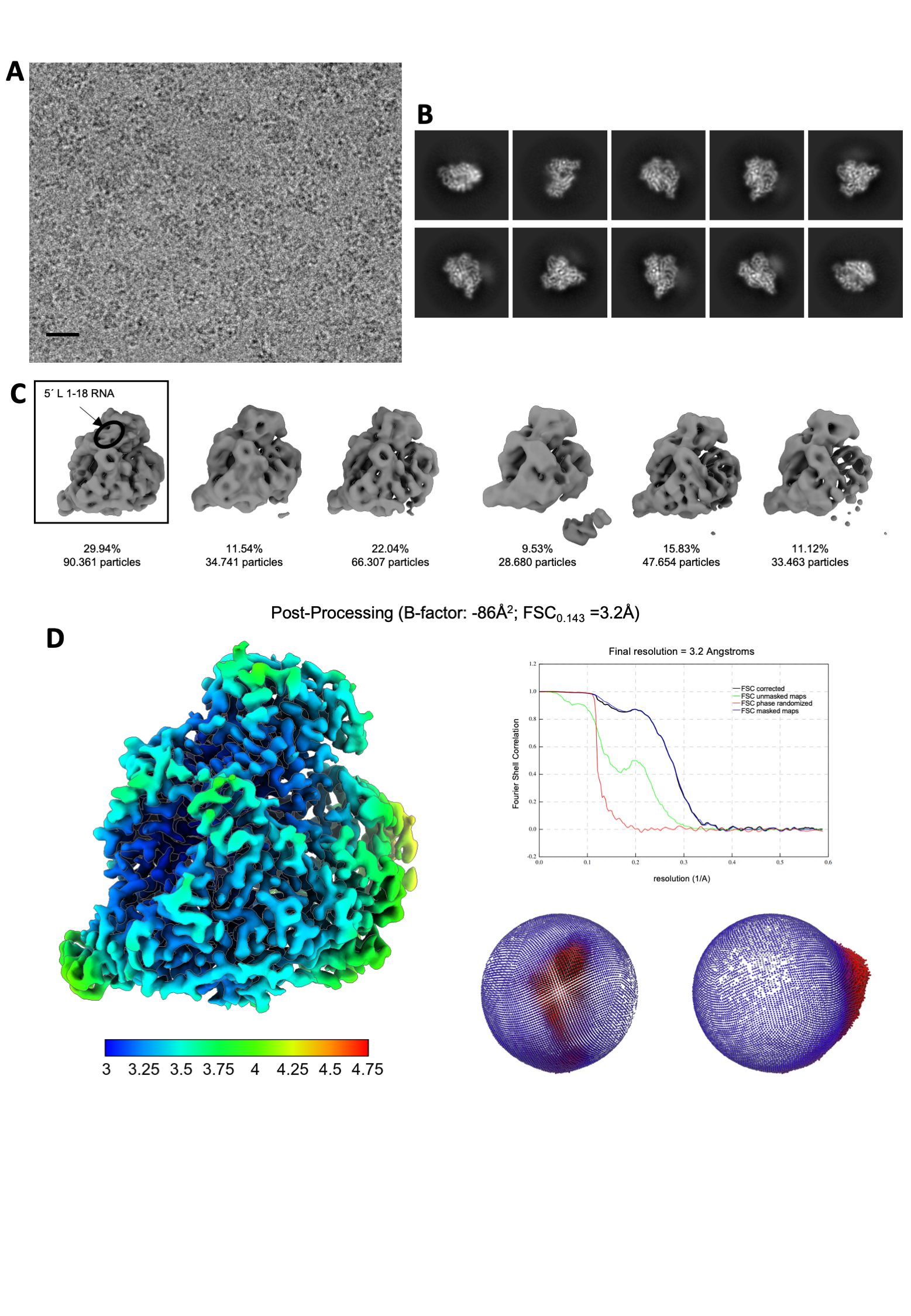

Supplement: S6 Fig — (A) Display of a representative micrograph after MotionCor2. The scale bar corresponds to 200 nm. (B) Representative 2D class averages. (C) 3D class averages of the 2nd 3D classification. The surrounded 3D class contains the particles selected for the last 3D reconstruction. The presence of the 5’ 1–18 RNA forming the hook is indicated. The percentage of particles and the corresponding number is indicated below each class (D) Final reconstruction is displayed. Electron density map is colored according to the local resolution. Fourier Shell Correlation curves (FSC) and angular distribution of particles used in the final reconstruction are displayed. (TIFF) [file ppat.1011533.s007.tiff]

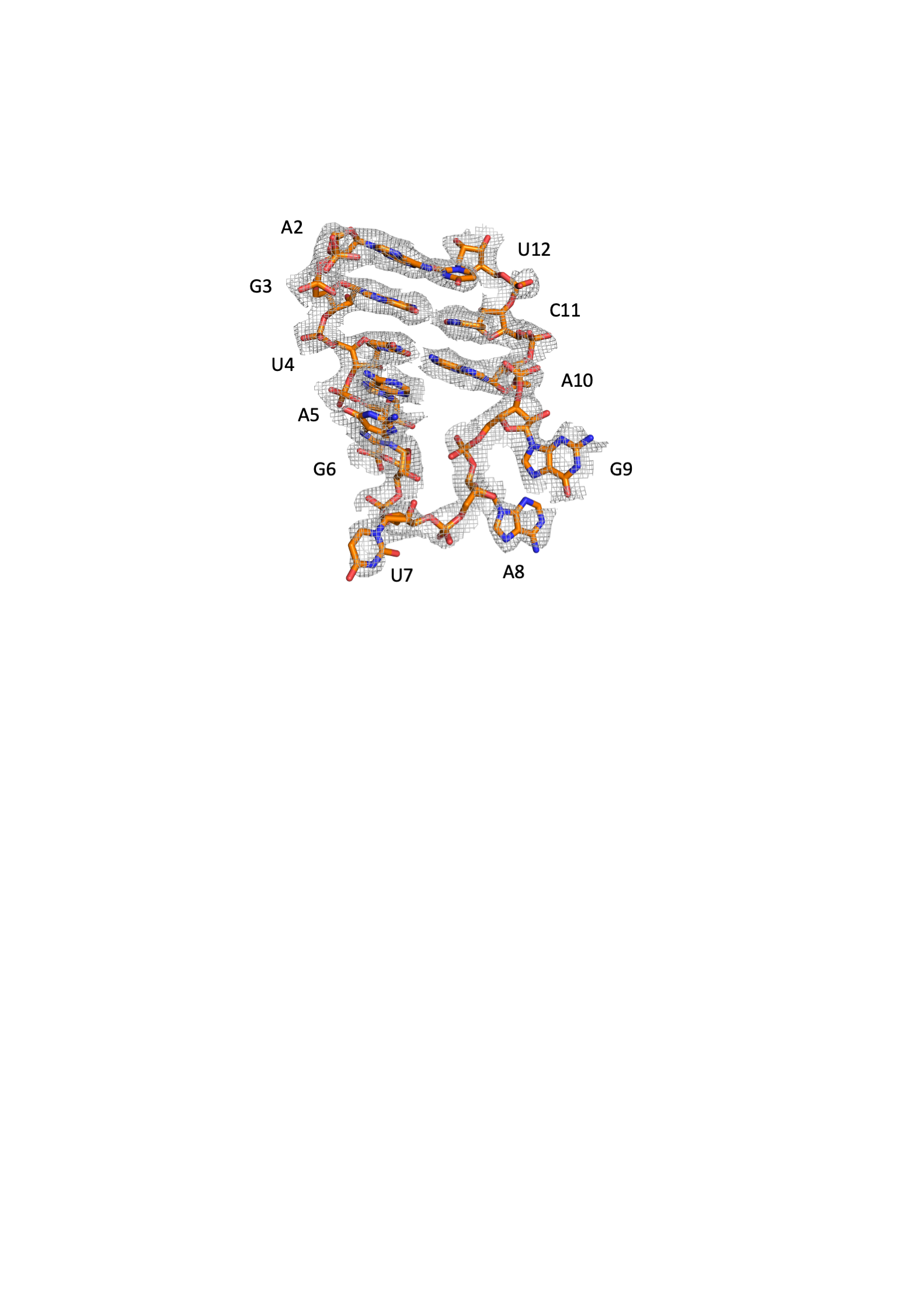

Supplement: S7 Fig — The RNA was built de novo in Coot [45] and was displayed as sticks in PyMol with the density displayed as mesh, contoured at 2.0 sigma within 1.5 Å of the displayed atoms. (TIFF) [file ppat.1011533.s008.tiff]

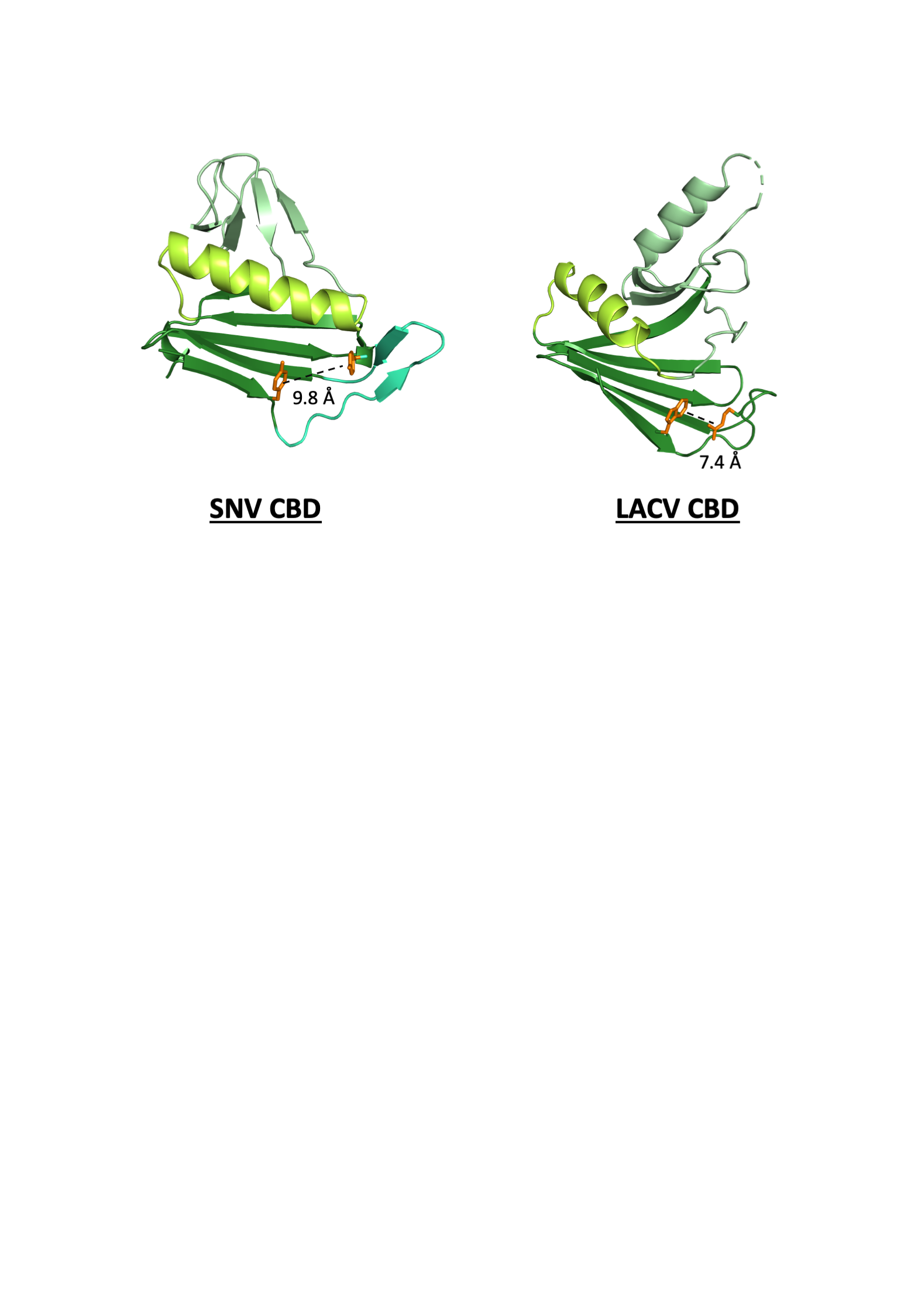

Supplement: S8 Fig — Structural comparison of the putative cap-binding domain within the C-terminal region of the hantavirus SNV L protein as predicted by AlphaFold2 [34] and the known structure of the phenuivirus LACV L cap-binding domain (from PDB 7ORL). The side chains that may be involved in forming the aromatic sandwich to bind the cap are shown as orange sticks. The black dotted line corresponds to the distance between these two side chains as measured in PyMol. (TIFF) [file ppat.1011533.s009.tiff]

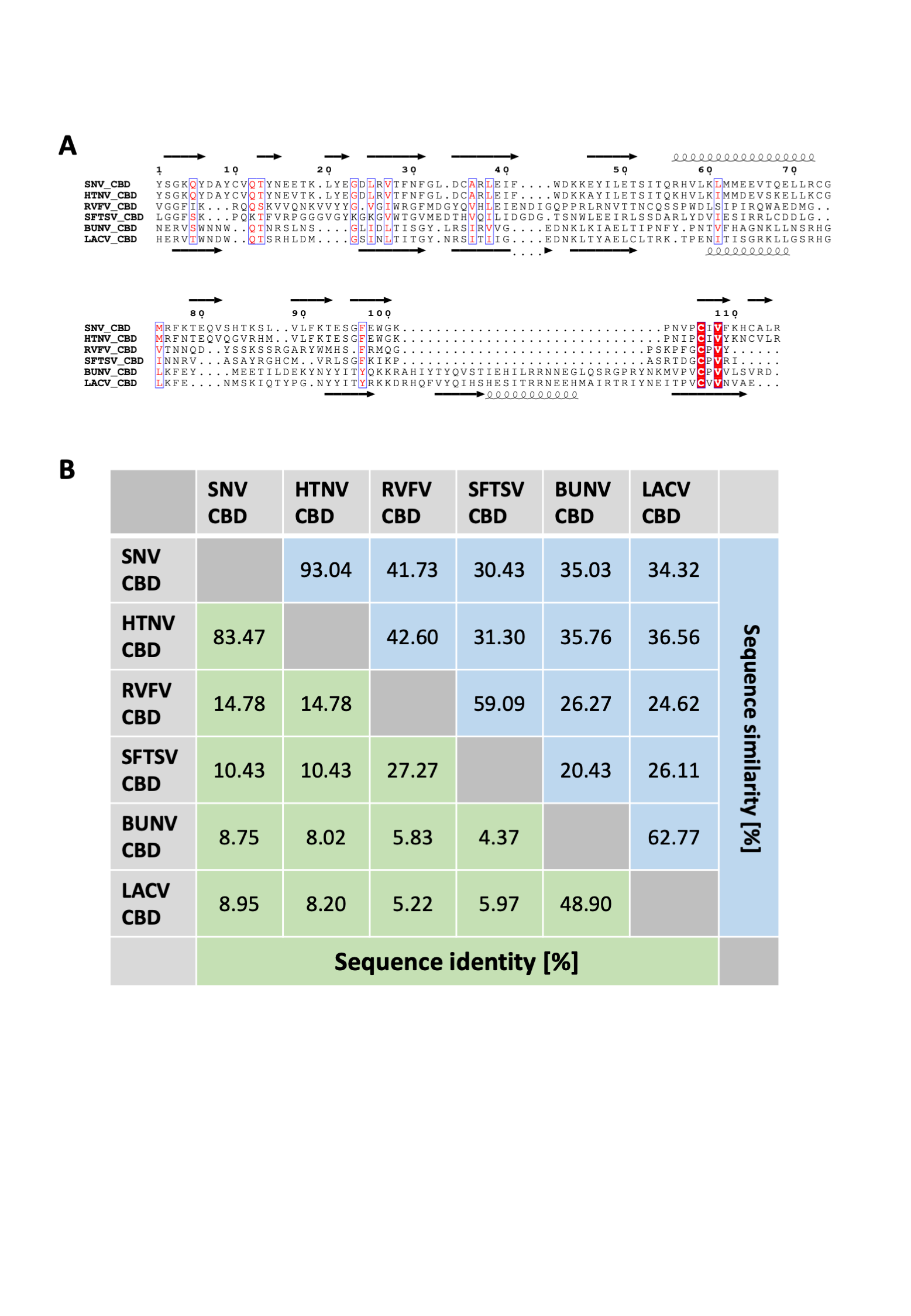

Supplement: S9 Fig — The L protein CBD sequences of hantaviruses Sin Nombre virus (SNV, KT885044.1) and Hantaan virus (HTNV, ABD28179.1), phenuiviruses Rift Valley fever virus (RVFV, A2SZS3) and Severe fever with thrombocytopenia syndrome virus (SFTSV, I0DF35), and peribunyaviruses Bunyamwera virus (BUNV, A0A0A7KU93), and La crosse virus (LACV, A5HC98) were aligned using Clustal Omega [47]. Manual adjustments were made and the alignment was visualized with ESPript 3 [48] Shown is a comparison of the secondary structure elements of the predicted SNV L CBD and LACV L CBD (PDB 6Z6G). (B) The sequence identity and similarity between the different CBD within the alignment shown in (A) were analysed using the Sequence Identity And Similarity tool (http://imed.med.ucm.es/Tools/sias.html). (TIFF) [file ppat.1011533.s010.tiff]
