# Supplementary material for: Structural and functional characterization of the Sin Nombre virus L protein
Source: PLoS Pathog. 2023 Aug 7;19(8):e1011533. doi: 10.1371/journal.ppat.1011533 (PMC10406178; doi:10.1371/journal.ppat.1011533)
Supplement: S1 Alignment — The L protein sequences of New World hantaviruses Sin Nombre virus (SNV, KT885044.1), Andes virus (ANDV, QRY27107.1), Bayou virus (BAYV, K7N869), and Black creek canal virus (BCCV, V5IVB1); Old World hantaviruses Hantaan virus (HTNV, ABD28179.1), Puumala virus (PUUV, ABN51178.1), Tula virus (TULV, A0A481S3H6), and Seoul virus (SEOV, A0A0B5JFL8), and peribunyaviruses Bunyamwera virus (BUNV, A0A0A7KU93), and La crosse virus (LACV, A5HC98) were aligned using Clustal Omega [47]. Manual adjustments were made and the alignment was visualized with ESPript 3 [48] Shown is a comparison of the secondary structure elements of SNV L and LACV L (PDB 6Z6G). (PDF) [file ppat.1011533.s011.pdf]

S1 Alignment. Alignment of *Hantaviridae* and *Peribunyaviridae* L protein sequences.

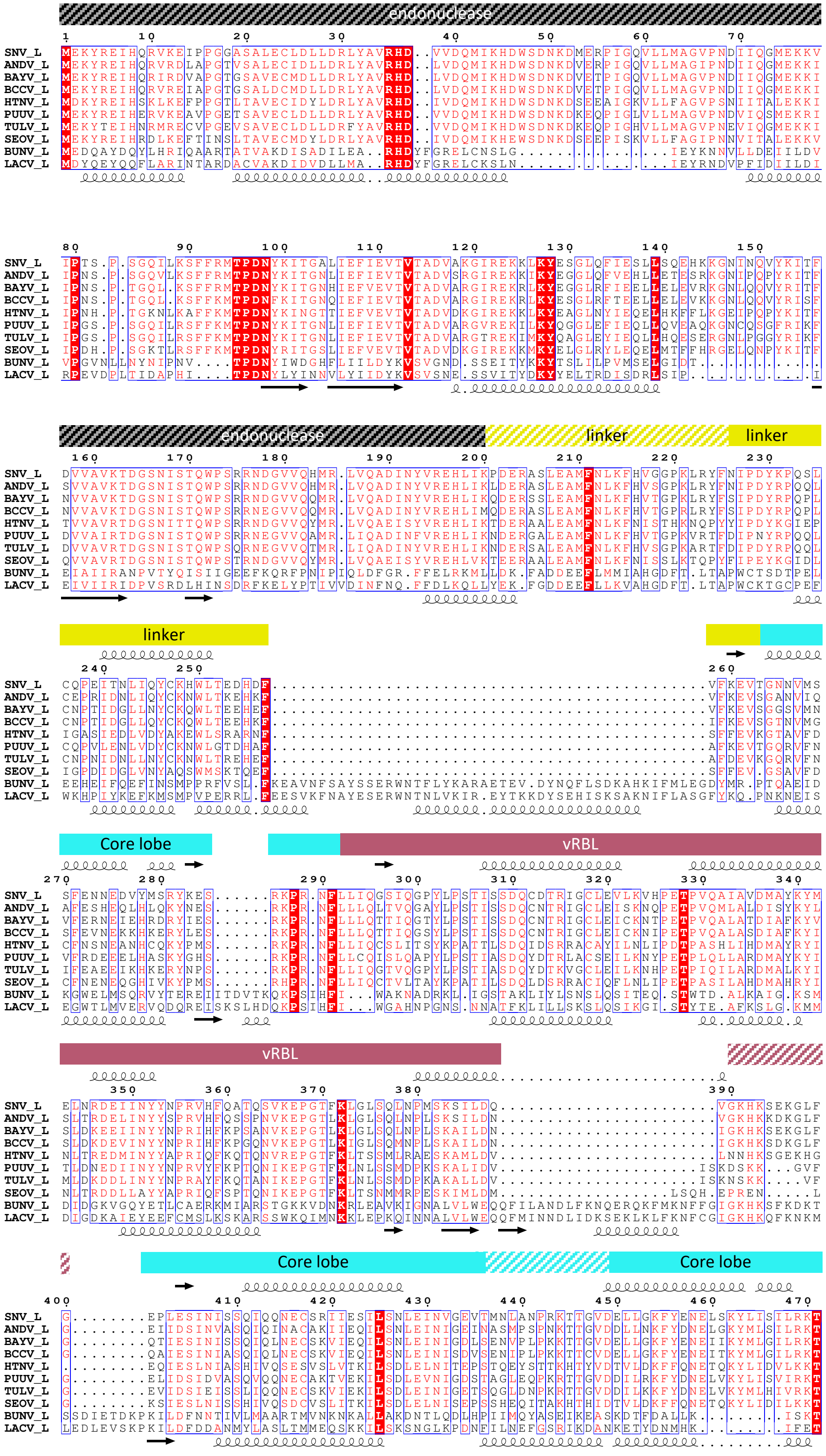

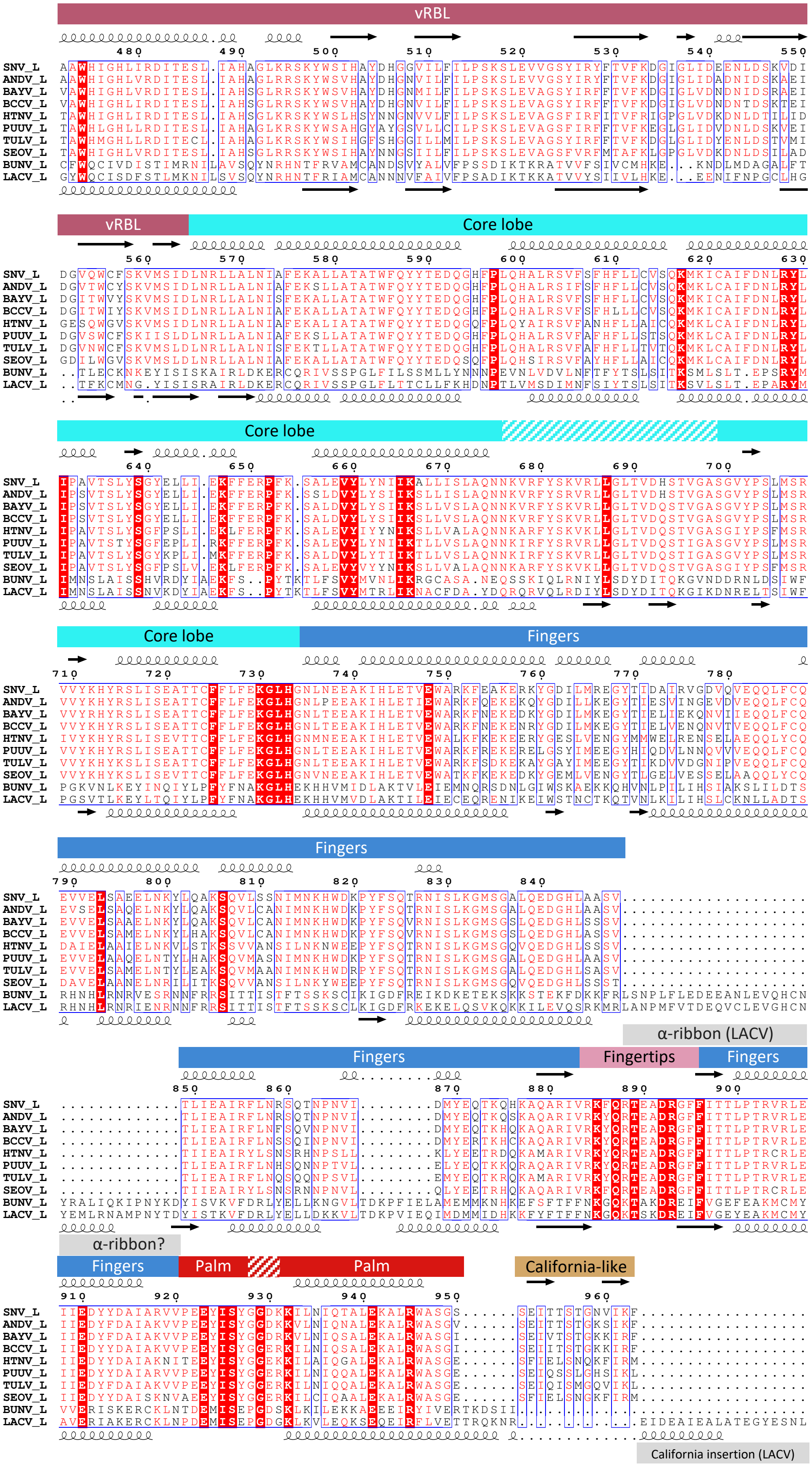

vRBL
vRBL
Core lobe
Core lobe
Core lobe
Fingers
Fingers
Fingers
Fingertips
Fingers
α-ribbon (LACV)
α-ribbon?
Fingers
Palm
Palm
California-like
California insertion (LACV)
SNV_L
ANDV_L
BAYV_L
BCCV_L
HTNV_L
PUUV_L
TULV_L
SEOV_L
BUNV_L
LACV_L

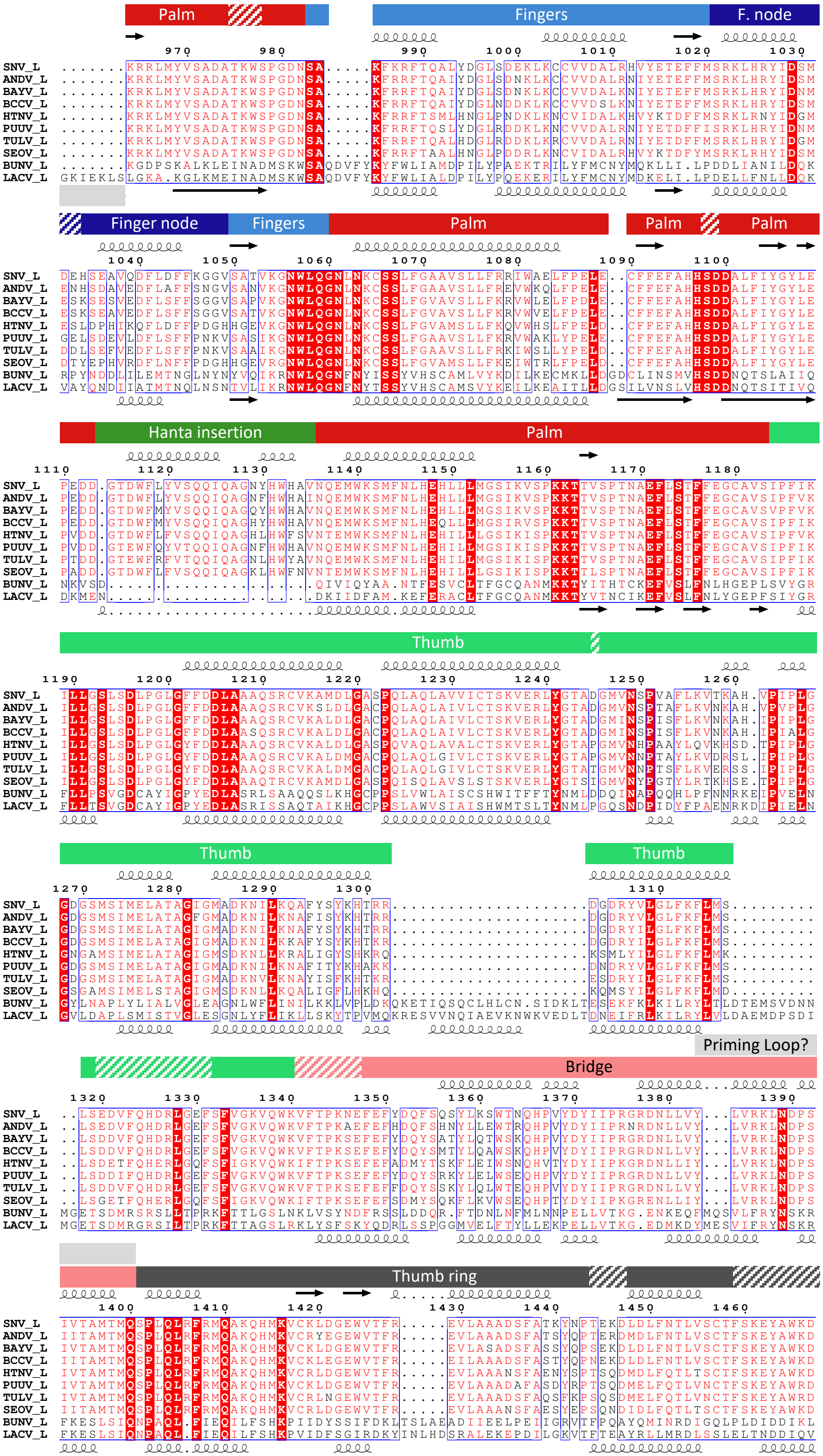
Palm
Fingers
F. node
Finger node
Fingers
Palm
Palm
Palm
Hanta insertion
Palm
Thumb
Thumb
Thumb
Priming Loop?
Bridge
Thumb ring
SNV_L
ANDV_L
BAYV_L
BCCV_L
HTNV_L
PUUV_L
TULV_L
SEOV_L
BUNV_L
LACV_L

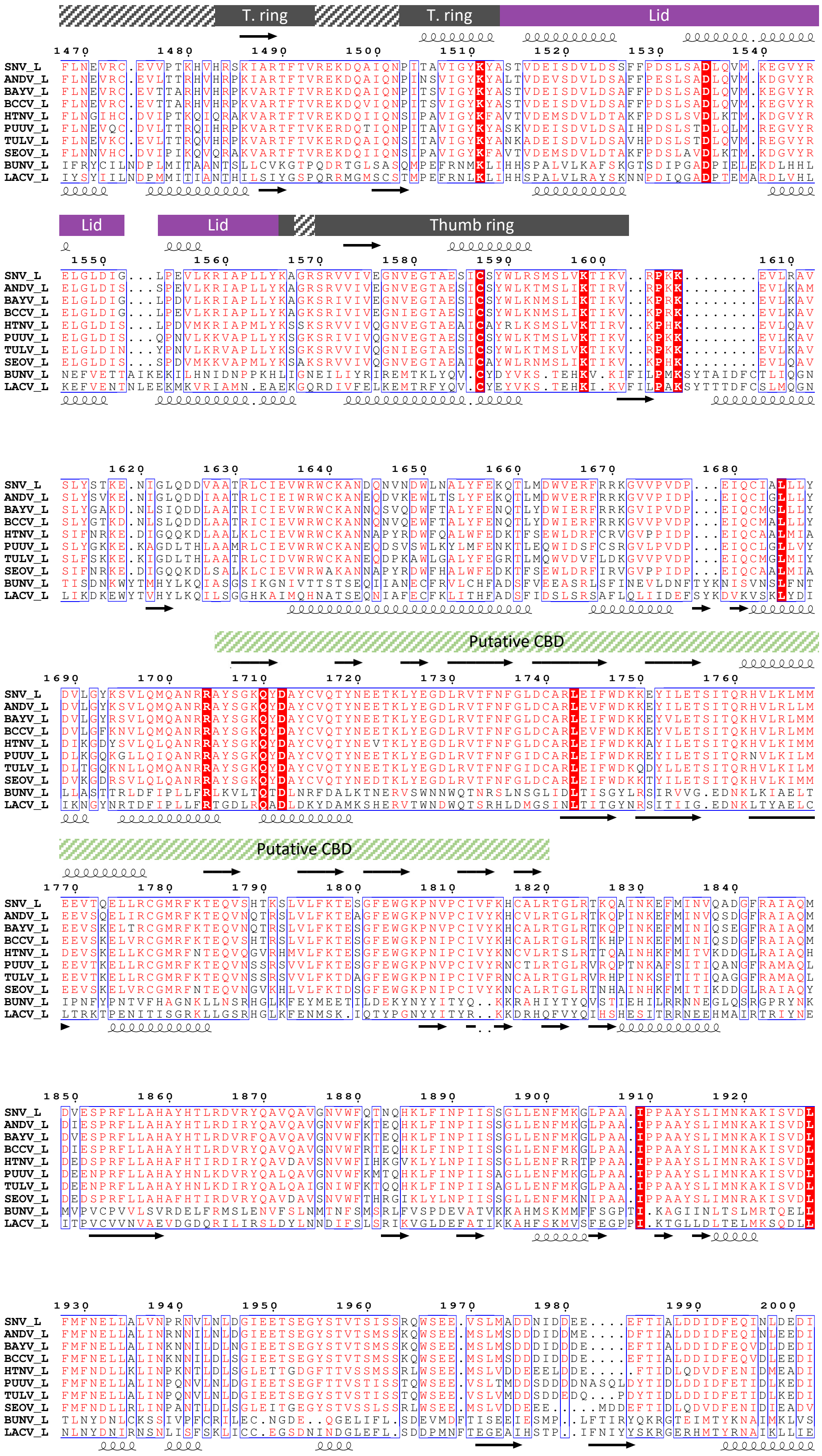
T. ring
T. ring
Lid
Lid
Lid
Thumb ring
Putative CBD
Putative CBD
SNV_L
ANDV_L
BAYV_L
BCCV_L
HTNV_L
PUUV_L
TULV_L
SEOV_L
BUNV_L
LACV_L

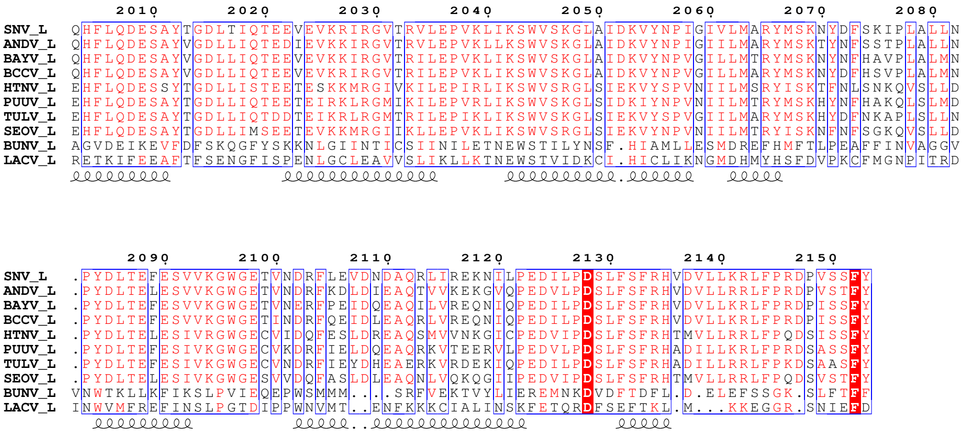

2010 2020 2030 2040 2050 2060 2070 2080
SNV_L QHFLQDESAYTGDLTIQTEEVEVKRIRGVTRVLEPVKLIKSWVSKGLAIDKVYNPIGIVLMARYMSKNYDFSKIPLALLN
ANDV_L QHFLQDESAYVGDLLIQTEDIEVKKIRGVTRVLEPVKLLKSWVSKGLAIDKVYNPIGIILMARYMSKTYNFSSTPLALLN
BAYV_L QHFLQDESAYVGDLLIQTEEVEVKKIRGVTRILEPVKLIKSWVSKGLAIDKVYNPVGILLMTRYMSKNYNFHAVPLALMN
BCCV_L QHFLQDESAYVGDLLIQTEEVEVKRIRGVTRILEPVKLIKSWVSKGLAIDKVYNPVGILLMARYMSKNYDFHSVPLALMN
HTNV_L EHFLQDESSYTGDLLISTEETESKKMRGIVKILEPIRLIKSWVSRGLSIEKVYSPVNIILMSRYISKTFNLSNKQVSLLD
PUUV_L EHFLQDESAYTGDLLIQTEETEIRKLRGMIKILEPVRLIKSWVSKGLSIDKIYNPVNIILMTRYMSKHYNFHAKQLSLMD
TULV_L EHFLQDESAYTGDLLIQTDDTEIKRLRGMTRILEPIKLIKSWVSKGLSIEKVYSPVGIILMARYMSKHYDFNKAPLSLLN
SEOV_L EHFLQDESAYTGDLLIMSEETEVKKMRGIIKLLEPVKLIKSWVSRGLSIEKVYNPVNIILMTRYISKNFNFSGKQVSLLD
BUNV_L AGVDEIKEVFDFSKQGFYSKKNLGIINTICSIINILETNEWSTILYNSF.HIAMLLESMDREFHMFTLPEAFFINVAGGV
LACV_L RETKIFEEAFTFSENGFISPENLGCLEAVVSLIKLLKTNEWSTVIDKCI.HICLIKNGMDHMYHSFDVPKCFMGNPITRD
2090 2100 2110 2120 2130 2140 2150
SNV_L .PYDLTEFESVVKGWGETVNDRFLEVDNDAQRLIREKNILPEDILPDSLFSFRHVDVLLKRLFPRDPVSSFY
ANDV_L .PYDLTELESVVKGWGETVNDRFKDLDIEAQTVVKEKGVQPEDVLPDSLFSFRHVDVLLRRLFPRDPVSTFY
BAYV_L .PYDLTEFESVVKGWGETVNERFPEIDQEAQILVREQNIQPEDILPDSLFSFRHVDVLLKRLFPRDPISSFY
BCCV_L .PYDLTEFESVVKGWGETINDRFQEIDLEAQRLVREQNIQPEDILPDSLFSFRHVDVLLKRLFPRDPISSFY
HTNV_L .PYDLTELESIVRGWGECVIDQFESLDREAQSMVVNKGICPEDVIPDSLFSFRHTMVLLRRLFPQDSISSFY
PUUV_L .PYDLTEFESIVKGWGECVKDRFIELDQEAQRKVTEERVLPEDVLPDSLFSFRHADILLKRLFPRDSASSFY
TULV_L .PYDLTEFESIVKGWGECVNDRFIEYDHEAERKVRDEKIQPEDILPDSLFSFRHADILLRRLFPKDSAASFY
SEOV_L .PYDLTELESIVKGWGESVVDQFASLDLEAQNLVQKQGIIPEDVIPDSLFSFRHTMVLLRRLFPQDSVSTFY
BUNV_L VNWTKLLKFIKSLPVIEQEPWSMMM....SRFVEKTVYLIEREMNKDVDFTDFL.D.ELEFSSGK.SLFTFF
LACV_L INWVMFREFINSLPGTDIPPWNVMT..ENFKKKCIALINSKFETQRDFSEFTKL.M...KKEGGR.SNIEFD
