# Supplementary material for: Structural and functional characterization of the Sin Nombre virus L protein
Source: PLoS Pathog. 2023 Aug 7;19(8):e1011533. doi: 10.1371/journal.ppat.1011533 (PMC10406178; doi:10.1371/journal.ppat.1011533)
Supplement: Attachment — Submitted filename: Rebuttal_Letter_PlosPath_Meier_etal2023_MR220523.pdf [file ppat.1011533.s012.pdf]

BNITM
Bernhard Nocht Institute for Tropical Medicine

Bernhard Nocht Institute for Tropical Medicine
Bernhard-Nocht-Str. 74, 20324 Hamburg, Germany

Dr. Maria Rosenthal
Group Leader
Department of Virology
Bernhard-Nocht-Str. 74
20359 Hamburg, Germany
Tel +49 40 285380-930
E-Mail rosenthal@bnitm.de

The Editorial Board
PLOS Pathogens

May 22nd 2023

Dear Editorial Board Members,

We thank you and the reviewers for their careful consideration of our manuscript **"Structural and functional characterization of the Sin Nombre virus L protein"**. Their suggestions have enabled us to make significant improvements to our manuscript. Please find below our responses (in italics) to the reviewers' comments together with the corresponding revisions that we made.

Reviewer #1: In this manuscript, Meier and colleagues establish an expression and purification protocol for the L protein with a K124A endonuclease mutation of Sin Nombre virus (SNV). They perform functional characterisation of the L protein using RNA-binding, endonuclease and polymerase activity assays. In the second part of the manuscript, they present a 3D model of the L protein core region containing the RdRp domain, in complex with 5' promoter RNA, obtained using single-particle cryo-EM. The authors compare their biochemical and structural observations with those for other segmented negative-sense RNA viruses (sNSVs).

Overall, this is an important addition to a growing body of literature on the structure and function of L proteins of sNSVs. The study highlights some interesting specificities of hantavirus L proteins such as the presence of the Hanta and California-like insertions in the palm domain of the RdRp. Generally, the manuscript is well-written and the data are clearly presented; for most parts the data support the conclusions but there are some issues that will need to be addressed.

Reviewer #2: This manuscript by Meier and coworkers examines the L protein polymerase of Sin Nombre hantavirus. Purification of the Sin Nombre virus L protein has proven problematic due to its highly active endonuclease activity, which suppresses protein expression. In this manuscript, the authors describe how they successfully purified the L protein by introducing a mutation into the endonuclease domain. The purified protein was analyzed in a series of biochemical assays that examined its endonuclease and RNA synthesis activities and the structure of the Sin Nombre virus L protein in association with RNA was determined. The manuscript adds to the growing body of literature regarding segmented negative strand RNA virus polymerases and provides valuable information that can be leveraged for future structure-informed functional studies. While the manuscript adds valuable new information to the field, some of the results from biochemical assays raised some questions that should be addressed.

Part II – Major Issues: Key Experiments Required for Acceptance

Reviewer #1:

1. Fig. 2. I find it surprising that the radiolabelled RNA is almost fully degraded but there is no obvious depletion of the fluorescently labelled RNAs even at the highest metal concentrations. These data would be consistent with a phosphatase contamination in the L

protein prep (could the authors include an SDS-PAGE of the L protein prep used?). The authors should check that the "endonuclease" activity they observe is sensitive to DPBA (as in the subsequent figure).

We have added an image of an SDS-PAGE analysis demonstrating the high purity of the L protein (Fig. S1). Additionally, we have repeated the radioactivity-based endonuclease assay with DPBA as a negative control (Fig. 2A) and did not observe any RNA degradation in this condition. We now managed to visualize degradation products in addition to the decreasing intensity of the substrate bands. However, both the radioactivity- and the fluorescence-based assays are suitable to evaluate the endonuclease activity and therefore possible inhibitors for therapeutic purposes in the context of the full-length L protein.

2. The data in Fig. S3. (not referred to in the manuscript) suggest that the observed "endonuclease" activity is specific to polyA but the significance of this is not discussed. Given that all these data were obtained with an endonuclease mutant I wonder about the significance of these data; they seem to add little to an otherwise strong manuscript.

   Thank you for pointing this out. This figure is now Fig. S4 and referred to in line 213. The endonuclease activity is not specific to polyA substrates but may be more efficient in this case, likely because some of the viral RNA is protected from degradation as it is specifically bound in separate pockets of the L protein or the active site (in case of 3' RNA), see line 213-215. We believe that for drug development purposes it is important to know that the endonuclease is active even in context of the mutated full-length L protein. The established system allows for the study of regulatory mechanisms of enzymatic activities in context of the full-length L. The hantavirus endonuclease seems to be highly active also in cells and for other bunyaviruses diverse mechanisms of activity control have been described, which are currently unknown for hantaviruses. In addition, our system would be applicable for the development of allosteric endonuclease inhibitor. We include an explanation on this in the manuscript: "This assay setup is particularly suited for the testing and development of allosteric endonuclease inhibitors and would also allow studies on the regulatory mechanisms of the endonuclease activity in context of the full-length L protein as observed for other L proteins [19, 20]."

3. Fig. 3. In the in vitro polymerase activity assay the authors use an 18 long template but observe mostly longer and heterogeneous products that are unlikely to represent authentic transcription products. This issue is addressed in the Discussion and in fact the authors present a more convincing result using a 3' phosphorylated RNA template (Fig. S4) where a major 18 nt product is observed with some longer products that can be explained by template switching. I wonder why the authors don't show this result in the Results section instead of data in Fig. 3 that are difficult to interpret. A further issue with the data in Fig. 3B is that the DPBA experiment lacks a positive control; also, DPBA seems to change the band pattern of products in the Mg2+ conditions but this is not commented on. Comparing band patterns in panel A and B I wonder whether the label for Mn2+ and Mg2+ is switched around. Panel A lacks size markers.

   We exchanged the figure and present now a new figure 3: The assay was repeated with the 3' phosphorylated (blocked) template and we ran all samples on the same gel to facilitate

comparison of the product bands. A size marker is now also shown. In addition, a comparison of the number of products using a 3'-phosphorylated or unmodified template is now shown in supplementary figure 5A.

Reviewer #2:

4. It would be helpful to show the purified polymerase on a Coomassie stained denaturing polyacrylamide gel to provide an indication of polymerase purity.

   An SDS-PAGE gel is now included, see comment 1 to reviewer #1

5. Fig. 2: Panel A does not appear to be consistent with panel B, lanes 1-5. In panel A, 40 mer poly A was efficiently cleaved by the endonuclease, whereas in B a 27 mer poly A showed minimal cleavage. It should be determined if this is due to the RNA length or the presence of the fluorescent tags.

   This difference was most likely due to the different assay setups. It is not trivial to get the right timing to visualize both the substrate bands losing intensity and the product bands gaining intensity. This differs between fluorescence- and radioactivity-bases assay conditions as well. The radioactivity-based assay was now repeated with a labeled PolyA27 substrate to improve comparability to the fluorescence-based assay and exclude that any differences were caused by different RNA lengths.

   It is possible that the degradation observed in Fig. 2 is due to a contaminating RNAse given that the activity is not ablated by the K124A substitution. Are there additional mutations that could be introduced to ascertain if the endonuclease activity that is observed is due to the SNV L protein? Alternatively, if DBPA has specificity for viral endonucleases perhaps this could be used to confirm the specificity of this cleavage.

   It has been described for ANDV (hantavirus) ENDO domain that this specific mutation only partially limits the enzymatic activity. We chose this mutation intentionally to reduce but not entirely abolish ENDO activity. The protein we used is very pure (see SDS-PAGE). Nevertheless, we added an experiment using DPBA to the ENDO reaction to confirm specificity of the reaction catalyzed by SNV L rather than other nucleases (Fig 2A).

6. Fig. 3: It appears that the data presented in Fig. 3A and Fig. 3B are inconsistent with each other. In panel A, 10 mM MnCl2 appears to completely inhibit RNA synthesis activity, whereas this is not the case in Fig. 3B. If this is because the panel B was exposed for much longer than panel A, this should be stated in the results section or legend. If there is another reason, an explanation should be provided.

   This is a detection problem originating from the differences of intensity of the bands. The gel in the second panel had been exposed much longer. We repeated the assay including the DPBA control directly on the same gel ensuring the same exposure of all samples (new Figure 3).

7. Fig. 3B: The pattern of RNA product bands is very different in the reactions that contained MgCl2 +/- DBPA. An explanation should be provided for these results.

Thank you for pointing this out. Indeed, this was due to a mistake in the composition of the lanes for this figure with a slight shift of the last lane. As explained above, the assay was repeated with all conditions on one gel to facilitate comparison of the different lanes.

8. Fig. 3 and subsequent RNA gels, there are products as long as 40 nt in length, although the template is only 18 nt. In the discussion section (lines 518-522) it is suggested that this is due to template switching. However, in Fig. 5C, there were bands of ~15 and 18 nt, even in reactions lacking GTP. It is difficult to conceive how the polymerase generates products of 15 and 18 nt in length by template switching in -GTP conditions. Is it possible that the RNA is not fully denatured? In addition, the discrepancy between the template and product length should be mentioned in the Results section.

   In our setup we use a high concentration of urea and ensure a constant high temperature (48-49°C) of the gel while running. Samples are denatured by boiling just before loading. We had this problem of not fully denatured RNAs for other projects a few years ago and since then adjusted our assay. We therefore consider it unlikely that these bands constitute incompletely denatured RNAs. Concerning the product sizes in the non-GTP condition, we have shown previously that bunyaviral polymerases are low fidelity enzymes and can substitute a nucleotide rather easily (biochemical and also structural evidence). In our hands, termination or stalling has been observed only when two NTPs directly following each other were missing in the reaction. The observed minor product bands are thus not surprising for us. We have now described the discrepancy between template length and products in lines 260-264 of the results section as requested.

Part III – Minor Issues: Editorial and Data Presentation Modifications

Reviewer #1:

9. Fig. 1 and line 143. These data are suggestive that the interaction of the L protein with the 5’ RNA is stronger than with the 3’ RNA but no affinity measurements were performed to be able to conclude that the interaction is “significantly stronger”. Please tone down the language.

   We toned down the language to “appeared to be stronger”: “In addition, the interaction of SNV L with the 5’ RNA appeared to be stronger than with the 3’ RNA.”

10. Fig. 4. Size markers are lacking.

    We now show size markers for all denaturing gels.

11. Line 322. Fig. S1A is referred to here; is this correct?

    Yes, this is correct (now Fig. S2A). We added an explanation in the respective paragraph.

12. Fig. 5. The authors suggest that there is a single binding site for the di/tri nucleotides (template sequence at the bottom of panel A). Due to the repetition of the AUC motif at the 3’ end these primers can all bind at multiple sites in the template; should this be considered?

    We included a table displaying the different possible annealing sites and the expected product lengths in the figure and also mention this in the text.

13. Fig. 8 and Fig. S7. These two figures could be joined as a single supplementary figure and perhaps discussed in the Discussion rather than being presented as Results.

    So far, no experimental structure of hantavirus cap-binding domain has been published and the quality of AlphaFold2 predictions is high enough to present this as a result as it supports the comparison with other L proteins. We therefore prefer to keep these data in the results section.

14. Fig. S1A. The legend refers to 3' RNA but 5' RNA is shown in the figure.

    Thank you for pointing this out, it has been corrected (now Fig. S2A).

15. Fig. S5C. The authors indicate only one class contains RNA. It would be informative to include particle numbers for these classes to give an idea of RNA occupancy.

    This information was added to the Figure.

16. Fig. S6. Include nt labels to orient the reader.

    Labels were included.

17. Table S1 and line 668. The table states about 500k particles were used in the final reconstruction; however in the methods 90,361 are mentioned. Please check.

    Thank you for this comment. The table was corrected to 90,361 particles.

18. Line 528. "extent"

    Changed.

19. Line 589. Presumably "300 mM NaCl".

    Thank you, this was corrected.

20. Lines 595 and 598. No "reaction" occurs in an electrophoretic mobility shift assay.

    Ok. We changed the wording.

21. Line 625. Please check that 0.5 mM GTP is correct; this would result in a very low ratio of 32P-GTP most likely not sufficient for detecting the RNAs

    Thank you for this comment, we corrected this mistake.

Reviewer #2:

22. Line 299-300 – "Taken together, our data suggest that the SNV L RdRp has a higher specificity for purines, indicative of a low RdRp fidelity". This is worded oddly. It would make more sense if it was written as "Taken together, our data suggest that the SNV L RdRp has a low specificity for pyrimidines, indicative of a low RdRp fidelity".

    Appreciated and changed.

23. Line 475: the inhibitory effect of the 5´ RNA was reduced in the presence of short primers. Could this be because the primer competed with 5´ RNA for binding to the 3´ RNA? Is the sequence of SNV different than for other viruses, do the 5´ and 3´ RNAs have a higher Tm?

    In this assay (Fig. 5B), primers were used at equimolar concentrations to 3' and 5' RNAs and not preincubated with 3' RNA. Although a competition is indeed possible, the highly

complementary 5' RNA is expected to form a much more stable duplex with the template than the short primers. This is now included in the discussion (lines 535-537). The RNA termini are overall conserved within the bunyavirus families but contain different numbers of mismatches within the first 20 nucleotides when comparing sequences of different families (reviewed recently by Malet et al. Plos Path, doi: 10.1371/journal.ppat.1011060). Indeed, the complementarity between the genomic RNA termini is rather high in the Hantaviridae with only two mismatches within the first 18 nucleotides. The GC content as one indicator of the overall melting temperature is highest in Arenaviridae, for which the observed phenomenon of 5'-based inhibition does not exist.

All models and cryo-EM maps have been uploaded to respective databases and will be released upon acceptance of the manuscript. All other data are included in the manuscript and its supplementary files.

In summary, we have responded to all comments and have modified the manuscript according to the reviewers' suggestions and editorial requirements. We thank the reviewers for the constructive criticism and hope that the revised version is now fully acceptable for publication in *PLOS Pathogens*.

Yours faithfully,

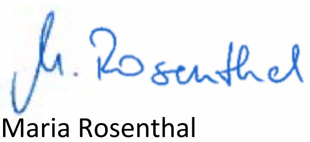

Maria Rosenthal
Maria Rosenthal
