# Supplementary material for: Structural and functional characterization of the Sin Nombre virus L protein
Source: PLoS Pathog. 2023 Aug 7;19(8):e1011533. doi: 10.1371/journal.ppat.1011533 (PMC10406178; doi:10.1371/journal.ppat.1011533)
Supplement: Attachment — Submitted filename: Rebuttal_ReRevision_PlosPath_Meier_etal2023_MR010723.pdf [file ppat.1011533.s013.pdf]

BNITM
Bernhard Nocht Institute for Tropical Medicine

Bernhard Nocht Institute for Tropical Medicine
Bernhard-Nocht-Str. 74, 20324 Hamburg, Germany

Dr. Maria Rosenthal
Group Leader
Department of Virology
Bernhard-Nocht-Str. 74
20359 Hamburg, Germany
Tel +49 40 285380-930
E-Mail rosenthal@bnitm.de

The Editorial Board
PLOS Pathogens

July 1st 2023

Dear Editorial Board Members,

Once again, we thank you and the reviewers for their careful consideration of our manuscript **"Structural and functional characterization of the Sin Nombre virus L protein"**. Please find below our responses (in italics) to the additional comments the reviewers made.

Reviewer #1: The authors have revised their paper satisfactorily addressing all the reviewers' comments. I have no further comments.

We thank the reviewer for their careful evaluation of our revised manuscript.

Reviewer #2: The reviewers' comments have been addressed and the conclusions appear to be sound. The only query I have is that on lines 210-213 it is stated that "we also detected degradation of the Cy5-labeled viral 3' and 5' RNAs 1-18 in substoichiometric RNA concentrations relative to the L protein, albeit with lower efficiency than the poly A substrate". However, looking at panel 2B, it is difficult discern a difference in cleavage of the polyA RNA versus the 3' 1-18 or 5' 1-18 RNAs. Revision of the accompanying text or an explanation would be helpful.

We thank the reviewer for their comment. This section also refers to the supplementary figure S4 in which the differences in RNA degradation between polyA and viral RNA are more obvious. We changed the section to the following: "Additionally, a fluorescence-based ribonuclease assay was established with various Cy5-labeled RNA substrates, in which we also detected degradation of the Cy5-labeled viral 3' and 5' RNAs 1-18 in sub-stochiometric RNA concentrations relative to the L protein (Fig. 2B), albeit with lower efficiency than the polyA substrate (Fig. S4)." We now separately refer to the respective figure.

We have responded to all reviewers' comments and have modified the manuscript according to the their suggestions. The revised version also follows the editorial requirements. We thank the reviewers for the constructive criticism and hope that this revised version is now fully acceptable for publication in *PLOS Pathogens*.

Yours faithfully,

M. Rosenthal

Maria Rosenthal
